# Mitochondrial phylogenomics provides conclusive evidence that the family Ancyrocephalidae is deeply paraphyletic

**DOI:** 10.1186/s13071-023-05692-6

**Published:** 2023-03-01

**Authors:** Cui-Lan Hao, Nian-Wen Wei, Yan-Jun Liu, Cai-Xia Shi, Kadirden Arken, Cheng Yue

**Affiliations:** College of Veterinary Medicine, Xinjiang Agricultural University, Urumqi, 830052 Xinjiang China

**Keywords:** Dactylogyridae, Dactylogyridea, *Dactylogyrus simplex*, *Dactylogyrus tuba*, Mitochondrial genome, Phylogeny, Paraphyly

## Abstract

**Background:**

Unresolved taxonomic classification and paraphyly pervade the flatworm class Monogenea: the class itself may be paraphyletic and split into Polyopisthocotylea and Monopisthocotylea; there are some indications that the monopisthocotylean order Dactylogyridea may also be paraphyletic; single-gene markers and some morphological traits indicate that the family Ancyrocephalidae is paraphyletic and intertwined with the family Dactylogyridae.

**Methods:**

To attempt to study the relationships of Ancyrocephalidae and Monopisthocotylea using a phylogenetic marker with high resolution, we sequenced mitochondrial genomes of two fish ectoparasites from the family Dactylogyridae: *Dactylogyrus simplex* and *Dactylogyrus tuba*. We conducted phylogenetic analyses using three datasets and three methods. Datasets were ITS1 (nuclear) and nucleotide and amino acid sequences of almost complete mitogenomes of almost all available Monopisthocotylea mitogenomes. Methods were maximum likelihood (IQ-TREE), Bayesian inference (MrBayes) and CAT-GTR (PhyloBayes).

**Results:**

Both mitogenomes exhibited the ancestral gene order for Neodermata, and both were compact, with few and small intergenic regions and many and large overlaps. Gene sequences were remarkably divergent for nominally congeneric species, with only *trnI* exhibiting an identity value > 80%. Both mitogenomes had exceptionally low A + T base content and AT skews. We found evidence of pervasive compositional heterogeneity in the dataset and indications that base composition biases cause phylogenetic artefacts. All six mitogenomic analyses produced unique topologies, but all nine analyses produced topologies that rendered Ancyrocephalidae deeply paraphyletic. Mitogenomic data consistently resolved the order Capsalidea as nested within the Dactylogyridea.

**Conclusions:**

The analyses indicate that taxonomic revisions are needed for multiple Polyopisthocotylea lineages, from genera to orders. In combination with previous findings, these results offer conclusive evidence that Ancyrocephalidae is a paraphyletic taxon. The most parsimonious solution to resolve this is to create a catch-all Dactylogyridae sensu lato clade comprising the current Ancyrocephalidae, Ancylodiscoididae, Pseudodactylogyridae and Dactylogyridae families, but the revision needs to be confirmed by another marker with a sufficient resolution.

**Graphical Abstract:**

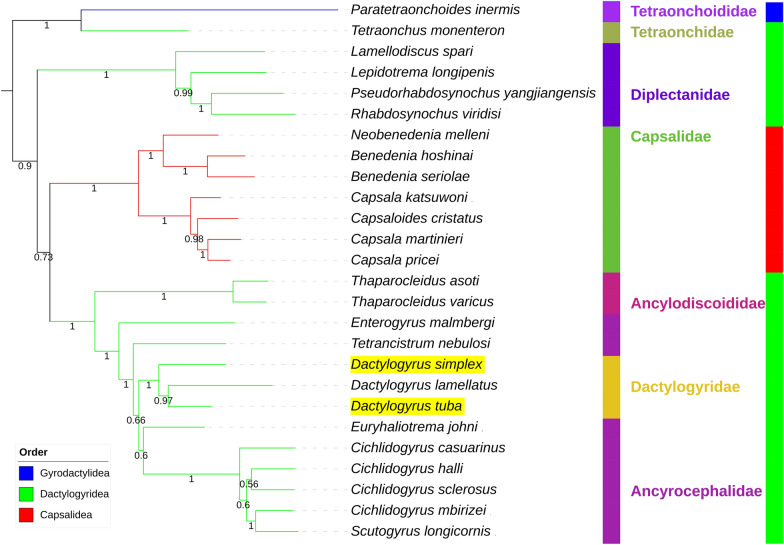

**Supplementary Information:**

The online version contains supplementary material available at 10.1186/s13071-023-05692-6.

## Background

Platyhelminthes (flatworms) is a phylum of largely parasitic animals that cause diseases in a broad range of host animal groups. The major radiation of parasitic flatworms represents the superclass Neodermata, divided into three classes: Monogenea, Trematoda and Cestoda. Monogenea comprises a large part of the flatworm diversity, but there are indications that this class might be paraphyletic and split into two independent subclasses: Polyopisthocotylea and Monopisthocotylea [1–4]. Monopisthocotylea is divided into five orders: Dactylogyridea, Capsalidea, Gyrodactylidea, Monocotylidea and Montchadskyellidea. Dactylogyridea Bychowsky, 1937, is a large order whose lower level taxonomy remains debated, which might be paraphyletic itself [5–7]. Currently, there are 11 valid families within the order, plus Ancyrocephalidae Bychowsky, 1937, whose status remains uncertain [8]. The status of Ancyrocephalidae has a long and complicated history. Initially, it was established as a subfamily (Ancyrocephalinae) of the large family Dactylogyridae Bychowsky, 1933 [9], and later it was elevated to the status of a family [10]. Following this, several subsequent studies failed to find support for this revision using both morphological and molecular (single locus) data, so its status remains unresolved [11–13].

Traditionally, most studies of monogenean phylogeny relied on morphology and single-locus molecular markers, both of which often have limited resolution. Monogenean parasites usually have few reliable morphological traits, and host-induced morphological variability is common [6, 14, 15]. Due to the small amount of information (phylogenetic signal) that they carry, single-locus molecular markers generally have too limited resolving power for deep phylogenies. Phylogenomic datasets offer a much greater resolution, but due to the limited availability of data, their application in Platyhelminthes had been limited so far to two nuclear genomic studies [16, 17] and several studies using complete mitochondrial genomes, e.g. [2, 18, 19]. The mitochondrial genome offers a much higher resolution than single-gene markers, as well as unilinear inheritance and (mostly) absence of recombination, so mitochondrial molecular markers were initially thought to be the ideal molecular marker for studying the evolutionary history of life on earth [20]. Despite these remarkable comparative advantages, mitogenomes are not a fool-proof marker, and in some cases they can produce artefactual results because of heterogeneity in base composition and evolutionary rates, introgression, mutational saturation, etc. [21–24]. Their applicability for phylogenetic studies is often further hampered by the scarcity of data. Indeed, whereas annotated mitogenomes are currently available for nine Ancyrocephalidae species, only one Dactylogyridae mitogenome has been sequenced so far: *Dactylogyrus lamellatus* [7]. This is insufficient to resolve the relationships between Dactylogyridae and Ancyrocephalidae using mitogenomic data.

Despite its putatively recent origin, *Dactylogyrus* (Dactylogyrinae subfamily) is a remarkably speciose (> 900 nominal species) genus of parasites of freshwater fishes with global distribution [25, 26]. To generate data needed to assess the relationship between Ancyrocephalidae and Dactylogyridae, as well as the relationships of Dactylogyridea and Monogenea, herein we sequenced two poorly studied *Dactylogyrus* species. *Dactylogyrus simplex* Bychowsky, 1936, is a small parasite that attaches itself to the gill lamellae of a broad range of fish hosts [27, 28]. Despite the almost global distribution (reported from Eurasia, North America and Africa), there are very few mentions of this species in scientific literature, and there are even fewer molecular data available: only two ITS1 (internal transcribed spacer 1) sequences in the GenBank. *Dactylogyrus tuba* Linstow, 1878, also attaches itself to the gills or nasal cavity of a wide range of Cyprinidae hosts, but predominantly from the Leuciscinae subfamily, and their distribution appears to be limited to Eurasia [26, 28–30]. This species is also rarely mentioned in the scientific literature, and in terms of available molecular data, there are only three ITS1 sequences in the GenBank.

## Methods

*Dactylogyrus simplex* was sampled from the host *Diptychus maculatus* Steindachner, 1866 (Cyprinidae), obtained from the Taxkorgan River (a tributary of the Yarkand River), Xinjiang, China (37°41′14″ N; 75°18′9″ E). *Dactylogyrus tuba* was sampled from the host *Leuciscus baicalensis* (Dybowski, 1874) (Leuciscinae) obtained from the Haba River (a tributary of the Irtysh River), Xinjiang, China (48°7′58″ N; 86°23′24″ E). Parasites were identified morphologically according to Gussev et al. [28] (Additional file 1: Figures S1 and S2) and molecularly using the ITS1 data. The *cox1* barcode identification did not produce any similarity to the available sequences. *Dactylogyrus simplex* exhibited 99.46% identity to the two available conspecific ITS1 sequences (MT476981 and MT476980). *Dactylogyrus tuba* exhibited 99.82% identity to the conspecific ITS1 sequence KJ605445 and 97.2% identity to the other two nominally conspecific sequences (AJ564157 and AJ564158).

DNA extraction, mitogenome amplification and sequencing (Sanger), as well as the sequence annotation and analyses, were conducted exactly as described before [31, 32]. ITS1 sequences were obtained using two primers designed by us based on available orthologues: D (5′-GGNGTCGATGAAGAACGCAG-3′) and B1 (5′-CGGATCCGAATCCTGGTTAG-3′). For the annotation of sequenced mitogenome, we selected the only other available congeneric mitogenome as the reference: *Dactylogyrus lamellatus* [7]. tRNAs were identified using ARWEN [33] and MITOS [34] programmes. ORF-Finder [35] was further used to search for genes resembling *atp8* in large noncoding regions of both mitogenomes. We downloaded all available Monopisthocotylea mitogenomes. As mitogenomes remain unavailable for Monocotylidea and Montchadskyellidea, we could only include representatives of three monopisthocotylean orders: Gyrodactylidea, Dactylogyridea and Capsalidea. To stabilize the topology as much as possible, we treated the entire Gyrodactylidea clade as the outgroup, as previous studies based on different types of data consistently supported it as a closely related but mutually monophyletic clade in relation to Dactylogyridea [4, 7, 18]. From this dataset, we removed all unannotated mitogenomes and left only one mitogenome per species. We also removed *Ancyrocephalus morgundae* (Ancyrocephalidae), because this mitogenome was incomplete (four PCGs missing: *cox2, nad2, nad4, nad4L*). After the pruning, there were 39 mitogenomes in the dataset (including the two new sequences). We also made minor taxonomic adjustments: Tetraonchidae is treated as a Gyrodactylidea family in the NCBI and Dactylogyridea family in WORMS, so we changed the taxonomic identity according to WORMS, as this database is updated more regularly [36]. PhyloSuite [37] was used to standardize the annotation, extract data, generate comparative tables and conduct phylogenetic analyses using several plug-in programmes. The TreeSuite function of PhyloSuite was used to infer the root-to-tip branch lengths, signal-to-noise ratio, relative composition variability score, treeness and long-branch scores [38, 39].

We used two mitogenomic datasets: (i) nucleotide sequences of 12 concatenated PCGs and two rRNA genes (NUC) and (ii) amino acid sequences of 12 concatenated PCGs (AAs). PCGs for the NUC dataset were first aligned using the codon mode and the accurate G-INS-i strategy in MAFFT [40] and alignments were then refined using MACSE, which is better at processing frameshift mutations [41]. rRNAs and AAs were aligned using only MAFFT (I-INS-i and G-INS-i strategies respectively). rRNAs were included to maximise the resolution. Genes were concatenated and partitioned by PhyloSuite (each gene in its own partition) and best-suited evolutionary models for partitions were inferred using another plug-in PhyloSuite programme: ModelFinder [42]. For phylogenetic reconstruction, we used three different algorithms: (i) maximum likelihood (ML) in IQ-TREE [43], (ii) Bayesian inference (BI) in MrBayes 3.2.6 [44] and (iii) CAT-GTR algorithm in PhyloBayes, designed to account for compositional heterogeneity [45]. ML analysis was run with 20,000 ultrafast bootstraps [46]. For the BI analysis, we used two parallel runs and 25% burn-in. For the ITS1 phylogenetic analyses, we downloaded all orthologous sequences belonging to Dactylogyridae, Ancyrocephalidae, Ancylodiscoididae and Pseudodactylogyridae. From other Dactylogyridea families, we retrieved representatives if they were available. Two Gyrodactylidae species were used as outgroups. We removed all sequences shorter than 800 bp, most of the conspecific sequences and all sequences that exhibited large deletions or missing data in the alignment. The final dataset comprised 67 species. We used the E-INS-i strategy in MAFFT to align them. iTOL [47] was used to visualise the phylogeny and architecture using files generated by PhyloSuite.

## Results

### Comparative mitogenomic architecture

**Fig. 1:**
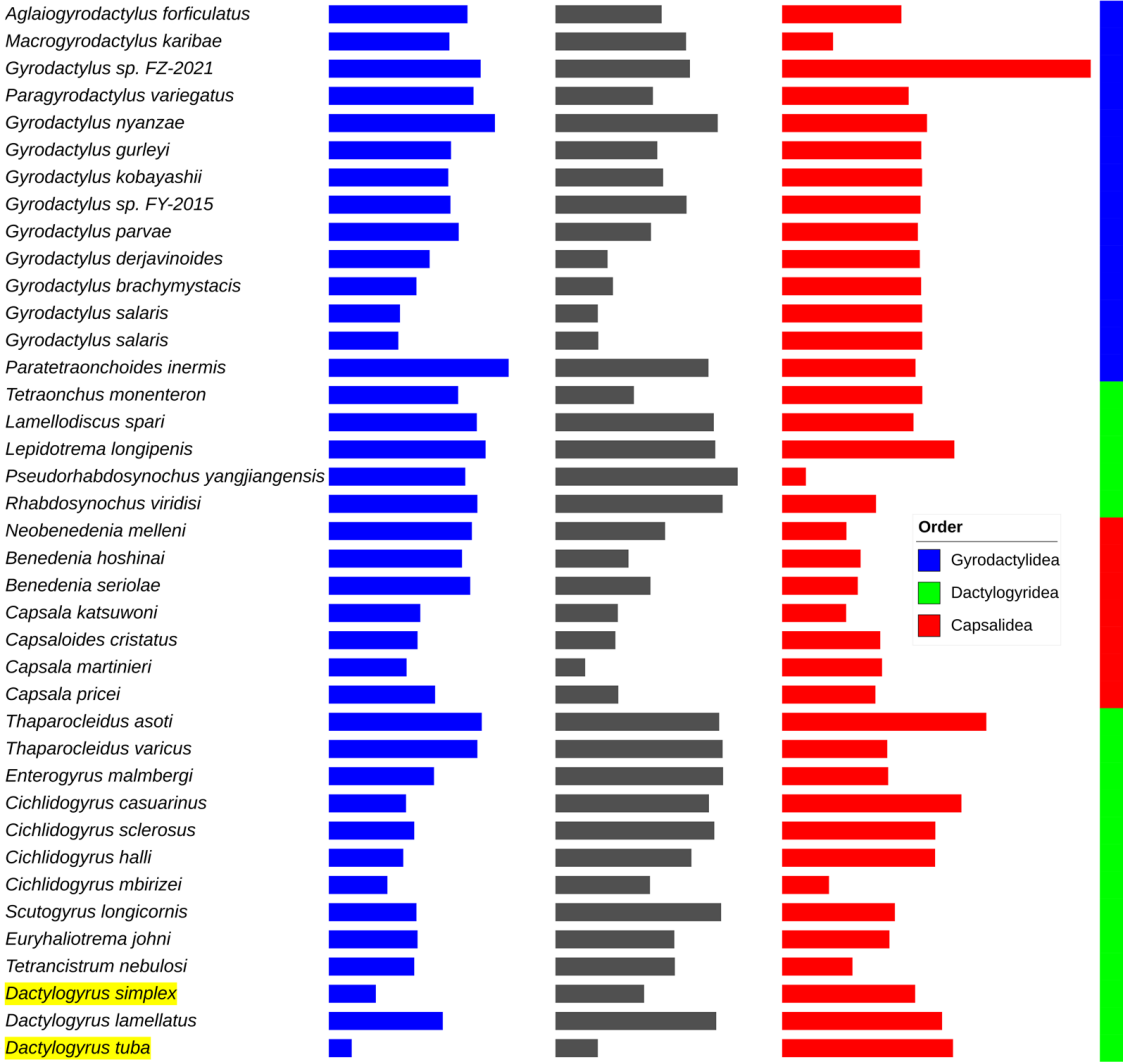
Comparative mitogenomic base composition and size in Monopisthocotylea. Bar charts show, from left to right: AT content (blue), GC skew (grey) and mitogenome size (red; 12,000 bases is set as 0). The order level taxonomic identity is shown to the right

At 14,648 bp in *D. simplex* and 15,406 bp in *D. tuba*, the two mitogenomes were average in size within the Monopisthocotylea dataset (Fig. 1, Additional file 2). Both comprised the standard neodermatan 36 genes (*atp8* missing from both), all of which are transcribed from the same strand. Both mitogenomes exhibited a single large (> 100 bp) NCR (likely to comprise the control region), but it was located in different places. Comparison with related species (Additional file 1: Figure S3) indicates that *D. tuba* exhibits the ancestral architecture, with the NCR located downstream from *nad5*, whereas *D. simplex* exhibits a derived architecture, with the NCR located between *cox1* and *rrnL*. The NCR was much larger in *D. tuba* (2,401 bp) than in *D. simplex* (1532 bp). We confirmed that *atp8* is not encoded in the two mitogenomes by searching both large NCRs for open-reading frames (ORFs). Both NCRs contained multiple putative ORFs, but none of them corresponded to the key characteristics of *atp8* genes identified in other Lophotrochozoa: starting with MPQM of MPHM motif and length between 38 to 56 AAs [48]. We also examined the identified ORFs encoded on the plus strand using the BLASTp suite, but none exhibited similarity to known genes. In other aspects, the two species exhibited an identical gene order, also common in other Neodermata (Table 1; Additional file 1: Figure S3). Compared to these two species, the congeneric *D. lamellatus* exhibits a transposition of the *trnL2* gene. Mitogenomes were compact in both species, with few and small noncoding regions and a relatively large number of overlaps, two of which were very large (Table 1). The smaller of the two overlaps comprised *trnF* and *trnM*, and it was almost perfectly conserved in both species (20 bp in *D. simplex* and 21 in *D. tuba*). The largest overlap of 28 bp was found between two PCGs: *nad4* and *nad4L*. Identity values between genes of these two nominally congeneric species were remarkably low (e.g. 74% for *cox1*), with only *trnI* exhibiting an identity value of over 80% (Table 1).

**Table 1:** Comparative table of mitogenomes of *Dactylogyrus simplex* / *Dactylogyrus tuba*. IGR stands for intergenic nucleotides, where negative values indicate overlaps, and bp is base pair

Gene	Position		Size	IGR	Codon		Identity
	From	To	bp	bp	Start	Stop	%
cox1	1/1	1563/1560	1563/1560		GTG/ATG	TAG/TAG	74.22
trnT	1569/1560	1635/1622	67/63	5/-1			52.24
NCR	1636/6388	3167/8788	1532/2401				25.18
rrnL	3168/1623	4110/2568	943/946				68.77
trnC	4111/2569	4172/2629	62/61				58.06
rrnS	4173/2630	4931/3344	759/715				69.96
cox2	4932/3345	5516/3918	585/574		ATG/ATG	TAA/T	66.5
trnE	5519/3979	5587/4041	69/63	2/60			63.77
nad6	5592/4045	6038/4491	447/447	4/3	ATG/ATG	TAA/TAG	52.35
trnY	6040/4495	6102/4556	63/62	1/3			78.12
trnL1	6107/4557	6174/4622	68/66	4/–			72.06
trnS2	6175/4623	6239/4689	65/67				70.15
trnL2	6245/4691	6308/4754	64/64	5/1			64.62
trnR	6311/4755	6375/4821	65/67	2/–			69.12
nad5	6378/4819	7928/6387	1551/1569	2/-3	ATG/GTG	TAA/TAA	51.39
trnG	7934/8789	7998/8853	65/65	5/–			77.27
cox3	7999/8854	8644/9499	646/646		ATG/ATG	T/T	67.96
trnH	8646/9500	8708/9562	63/63	1/–			71.88
cytb	8714/9565	9785/10641	1072/1077	5/2	ATG/ATG	T/TAG	70.29
nad4L	9796/10641	10,041/10889	246/249	10/-1	ATG/GTG	TAG/TAA	63.45
nad4	10,014/10862	11,237/12079	1224/1218	-28/-28	ATG/GTG	TAA/TAA	52.85
trnQ	11,244/12082	11,304/12143	61/62	6/2			74.19
trnF	11,303/12142	11,379/12220	77/79	-2/-2			75
trnM	11,360/12200	11,425/12262	66/63	-20/-21			74.24
atp6	11,431/12264	11,940/12773	510/510	5/1	ATG/ATG	TAG/TAG	60.78
nad2	11,957/12775	12,796/13593	840/819	16/1	ATG/GTG	TAG/TAG	47
trnV	12,802/13593	12,864/13653	63/61	5/-1			68.25
trnA	12,872/13654	12,933/13716	62/63	7/–			77.78
trnD	12,940/13716	13,005/13780	66/65	6/-1			63.64
nad1	13,006/13781	13,887/14668	882/888		ATG/ATG	TAG/TAG	65.2
trnN	13,892/14674	13,950/14731	59/58	4/5			68.85
trnP	13,959/14738	14,024/14803	66/66	8/6			75.76
trnI	14,024/14803	14,090/14869	67/67	-1/-1			92.54
trnK	14,093/14870	14,154/14932	62/63	2/–			76.56
nad3	14,158/14933	14,496/15280	339/348	3/–	ATG/ATG	TAA/TAG	58.91
trnS1	14,512/15281	14,569/15337	58/57	15/–			79.31
trnW	14,575/15340	14,642/15402	68/63	5/2			60.29

The two newly sequenced mitogenomes exhibited exceptionally low A + T base content (*D. tuba* = 54%, *D. simplex* = 58.4%) compared to other Dactylogyridea species (60–78.4%; Fig. 1, Additional file 2). The T content was exceptionally low in *D. tuba* (29.4%) and very low in *D. simplex* (34%). In comparison, in the rest of the dataset, it ranged between 35.1 and 47.2%. Regarding A content, the two species were merely at the bottom of the range in the dataset, but both species had the highest C and G content in the entire dataset. Similarly, both species had exceptionally low (in absolute terms) AT skews, calculated as (A—T)/(A + T), in the dataset: − 0.087 in *D. tuba* and − 0.164 in *D. simplex* (dataset range = − 0.087 to 0.338). Correspondingly, *D. tuba* had the lowest GC skew, calculated as (G − C)/(G + C) in the dataset (0.085), whereas the GC skew of *D. simplex* was merely in the lowest quartile (0.18; dataset range = 0.085 to 0.372).

### Mitochondrial phylogenomic analyses

**Fig. 2:**
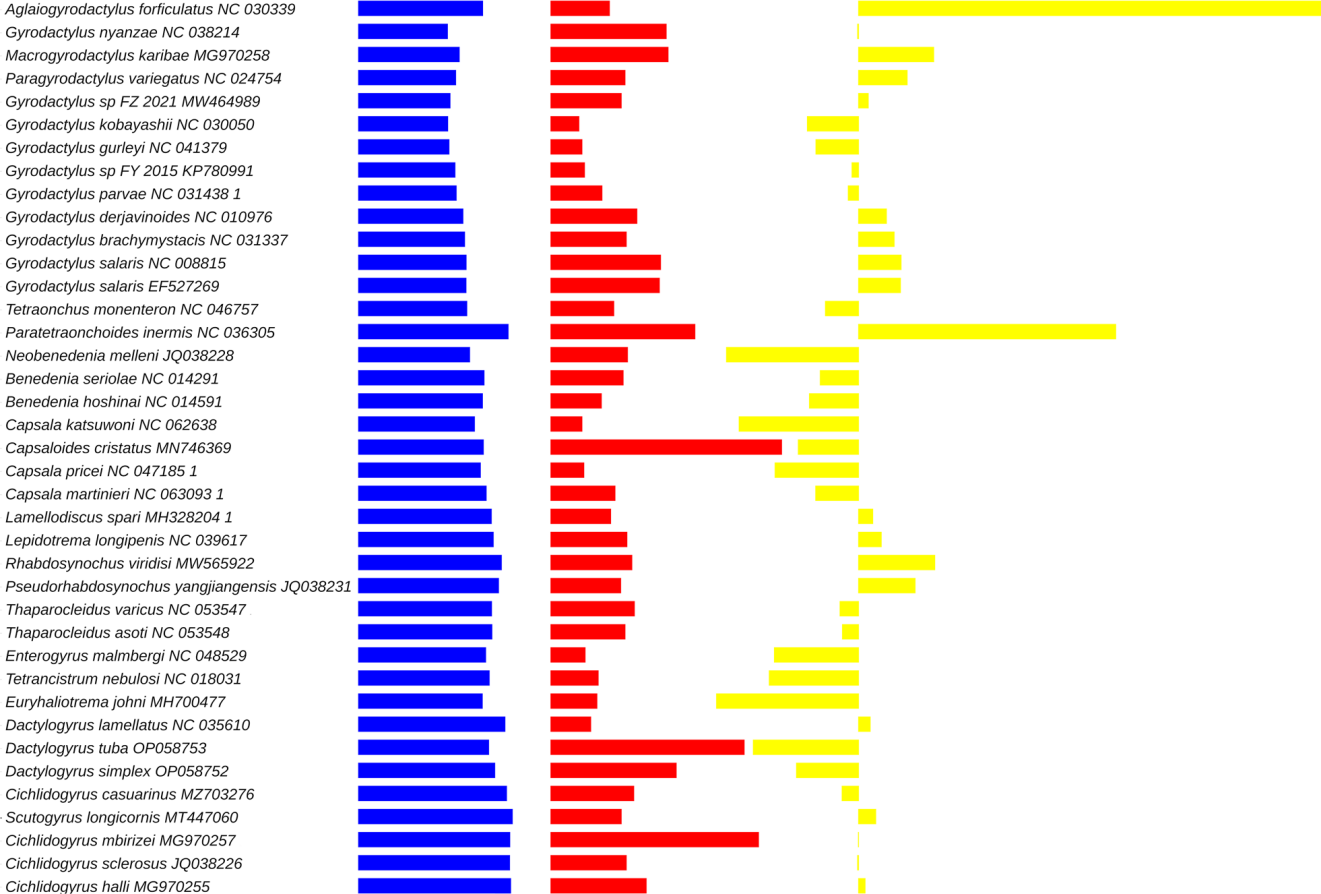
The root-to-tip branch lengths (blue), relative composition variability (red) and long-branch scores (yellow) in the mitogenomic Monopisthocotylea dataset

The concatenated NUC alignments of all 39 species in the dataset failed the compositional homogeneity test, whereas only ten species passed the test in the AA dataset. After *Capsaloides cristatus* (0.37) and *Cichlidogyrus mbirizei* (0.33), *D. tuba* (0.31) exhibited the third-highest relative composition variability in the dataset. Despite the unusual base composition, the two newly sequenced species had some of the lowest long-branch scores in the dataset (− 7.97 and − 4.70). The highest values were exhibited by *Aglaiogyrodactylus forficulatus* (34.95) and *Paratetraonchoides inermis* (19.48) (Fig. 2). The longest root-to-tip branch lengths were exhibited by several *Cichlidogyrus* and *Scutogyrus* species, along with *Paratetraonchoides inermis*, with the three *Dactylogyrus* species exhibiting relatively high values within the dataset. Signal-to-noise ratio was 2.05 and the treeness was 0.29 (the numbers refer to the BI_NUC tree, but overall results were similar across different trees).

The BI analyses were run until the average standard deviation of split frequencies (ASDSF) was stationary and < 0.01 (a very good indication of convergence according to MrBayes manual). Both BI analyses converged; for the NUC dataset, the final ASDSF was 0.0067 after 500,000 MCMC steps, and for the AA dataset, the final ASDSF was 0.0066 after 1,000,000 MCMC steps. PhyloBayes analysis (CAT-GTR) met the conditions for an acceptable run: AA maxdiff = 0.11 and NUC maxdiff = 0.22 (maxdiff < 0.3 indicates an acceptable run and maxdiff < 0.1 indicates a good run).

**Fig. 3:**
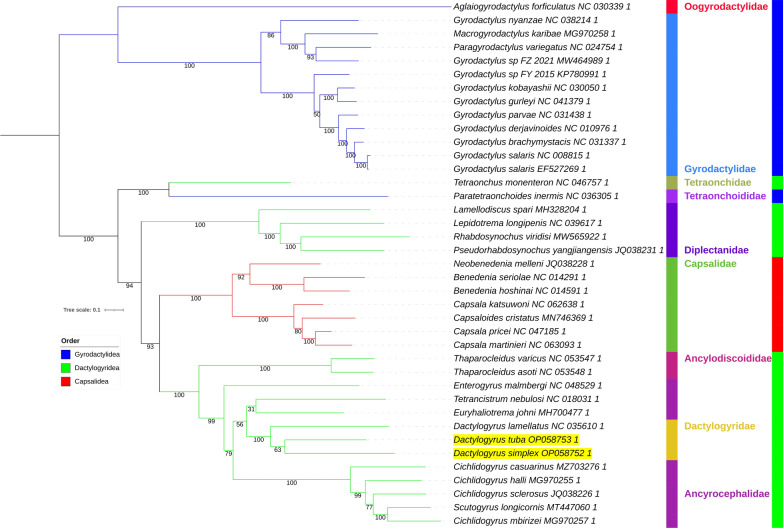
AAs_ML mitochondrial phylogenomic analysis of the Monopisthocotylea. The AA dataset comprises concatenated (partitioned) amino acid sequences of 12 PCGs. IQ-TREE was used to conduct the maximum likelihood (ML) analysis. Bootstrap support is shown next to the nodes. The family level taxonomy is shown to the right. Orders are indicated by a coloured strip and coloured branches, with the legend included in the figure. The two newly sequenced species are highlighted by a yellow background, and Gyrodactylidea is the outgroup

**Fig. 4:**
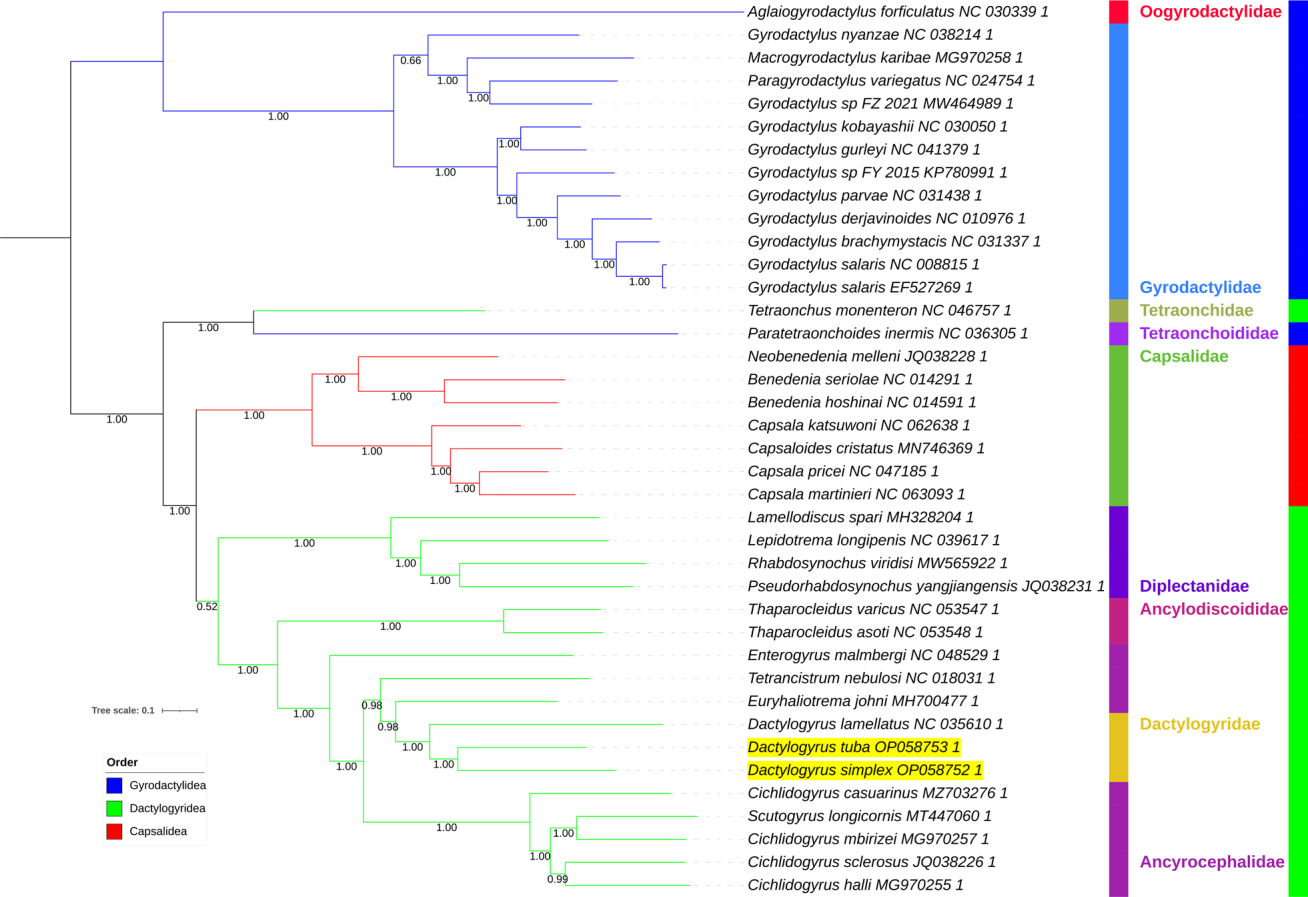
The NUC_BI mitochondrial phylogenomic analysis of the Monopisthocotylea. The NUC dataset comprised concatenated (partitioned) nucleotide sequences of 12 PCGs and 2 rRNAs. MrBayes was used to conduct the Bayesian Inference (BI) analysis. Posterior probability values are shown next to the nodes. For other details, see Fig. 3

**Fig. 5:**
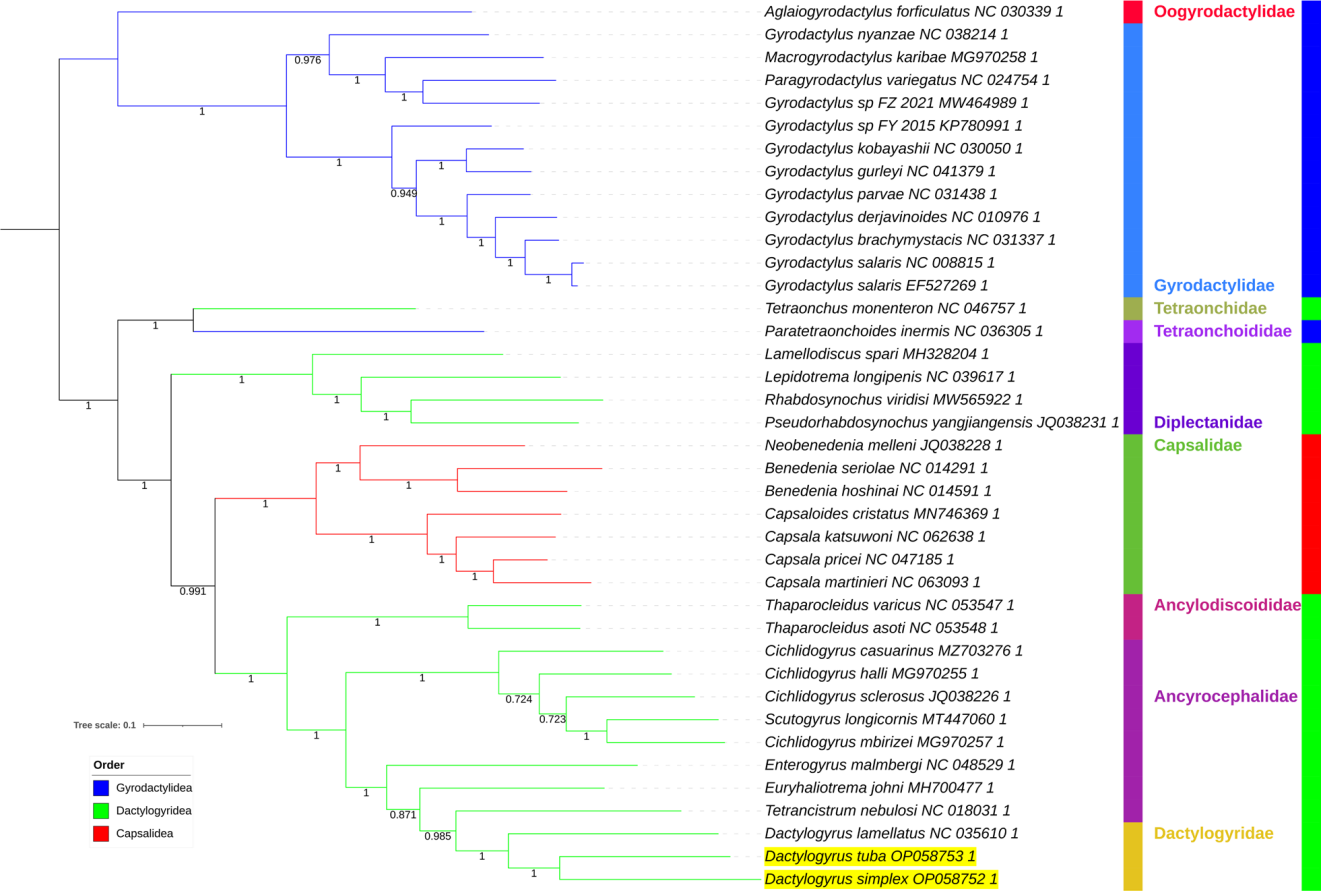
AAs_BI mitochondrial phylogenomic analysis of the Monopisthocotylea. For other details, see Figs. 3 and 4

**Fig. 6:**
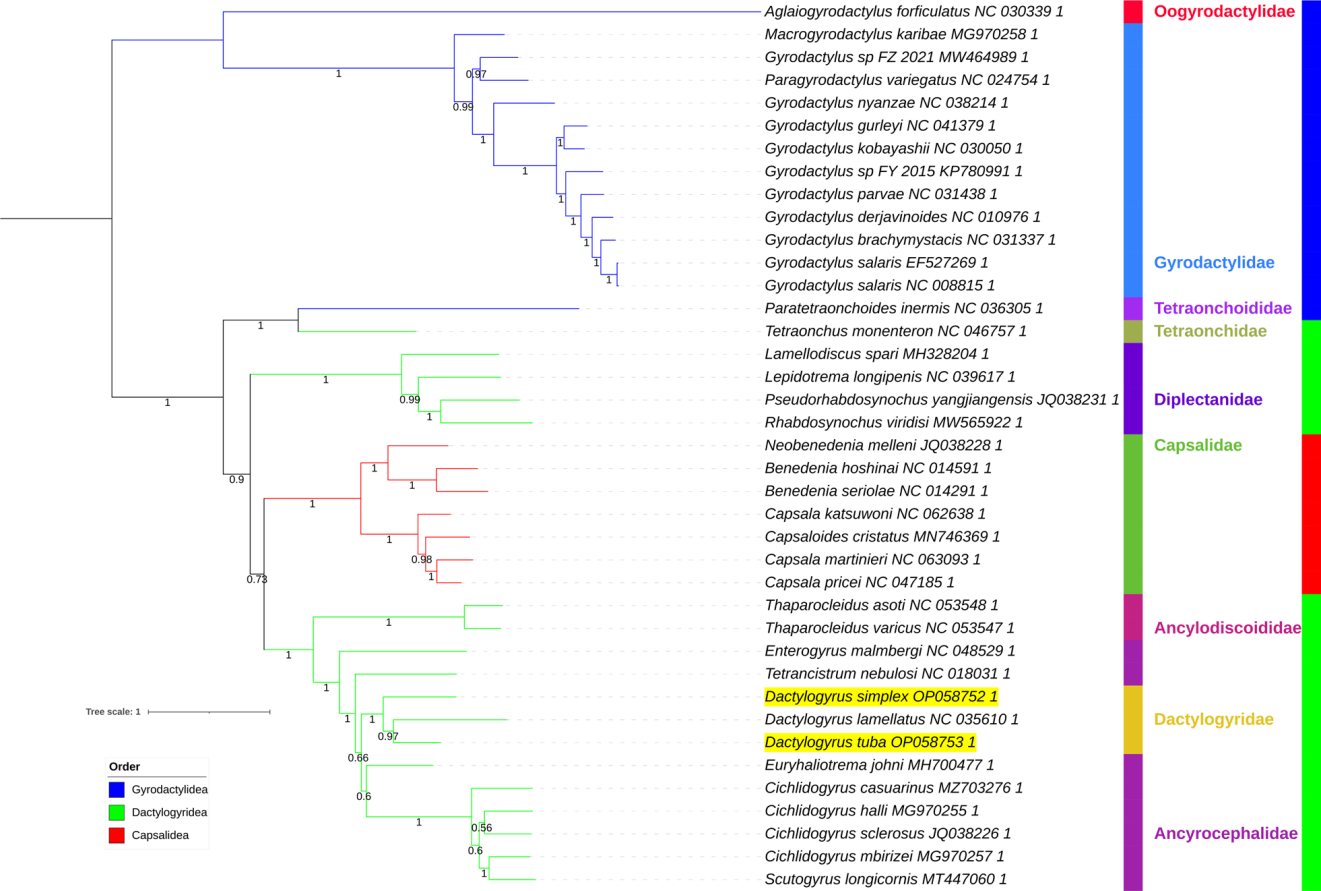
NUC_CAT-GTR mitochondrial phylogenomic analysis of the Monopisthocotylea. PhyloBayes (CAT-GTR algorithm) was used to conduct the analysis, and the datast was not concatenated. Posterior probability values are shown next to the nodes. For other details, see Figs. 3 and 4

**Fig. 7:**
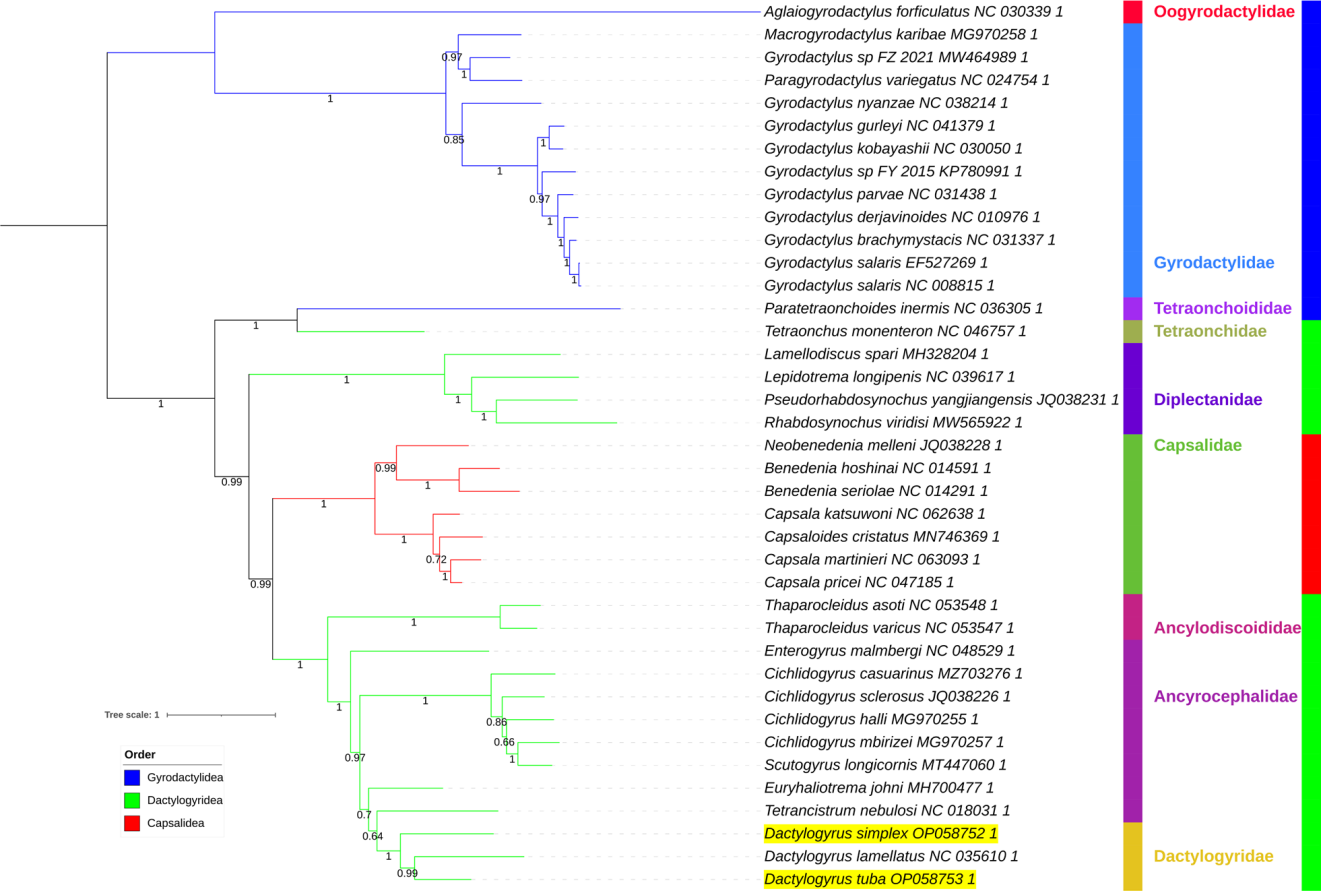
AAs_CAT-GTR mitochondrial phylogenomic analysis of the Monopisthocotylea. For other details, see Figs. 3, 4 and 6

All six analyses produced unique topologies (Figs. 3, 4, 5, 6, 7). As the two ML analyses differed only in minor topological details in the Gyrodactylidae clade and relationships between *Scutogyrus* and *Cichlidogyrus*, only the AA tree is shown (Fig. 3), whereas the NUC tree is available in Additional file: Figure S4. We also tested whether the inclusion of two rRNA genes affected the topology, so we removed the two genes and conducted an ML analysis using only the 12 PCGs. The topology was unaffected, but support values for some nodes differed: in some cases they were higher and in some lower (Additional file 1: Figure S5). Overall, there was no effect on the main conclusions. *Dactylogyrus* was monophyletic in all analyses, with *D. tuba* and *D. simplex* being sister species in ML and BI analyses. However, in both CAT-GTR analyses, *D. tuba* and *D. lamellatus* were sister species. Support values for this node were high in all analyses (≥ 97%), apart from the ML_AA tree, where it was 63% (Fig. 3). Dactylogyridae (monophyletic) were nested within the Ancyrocephalidae (paraphyletic) in all six analyses. All analyses aside from NUC_BI (all ≥ 98%) produced some support values < 90% in the Dactylogyridae + Ancyrocephalidae clade. The Dactylogyridae + Ancyrocephalidae clade was monophyletic and exhibited a sister-group relationship to Ancylodiscoididae in all analyses (with high support). The order Dactylogyridea was rendered paraphyletic by Capsalidea nested within the clade in all analyses except NUC_BI, where Capsalidea and Gyrodactylidea were sister groups (Fig. 4). Node support varied between high and low among the analyses. Disregarding Tetraonchidae, Diplectanidae was resolved as the basal (sister group to the rest of the clade) radiation of Dactylogyridea in all topologies aside from the NUC_BI. Both Dactylogyridea and Gyrodactylidea were rendered paraphyletic by Tetraonchidae and Tetraonchoididae forming a clade at the base of the Dactylogyridea. This clade was resolved with high confidence in all analyses. The Gyrodactylidae clade was not stable either. There were two main topologies regarding the position of *Gyrodactylus nyanzae*. In one topology, the Gyrodactylidae family was divided into two clades, with *G. nyanzae* clustering with *Paragyrodactylus* and *Macrogyrodactylus* (ML_AAs, both BI analyses, NUC_CAT-GTR). In the other topology (ML_NUC, AAs_CAT-GTR) *G. nyanzae* was resolved at the base of the main *Gyrodactylus* clade. *Gyrodactylus* was further rendered paraphyletic by *Gyrodactylus* sp. (MW464989) clustering closer to *Paragyrodactylus* and *Macrogyrodactylus* than to the rest of the nominally congeneric species.

**Fig. 8:**
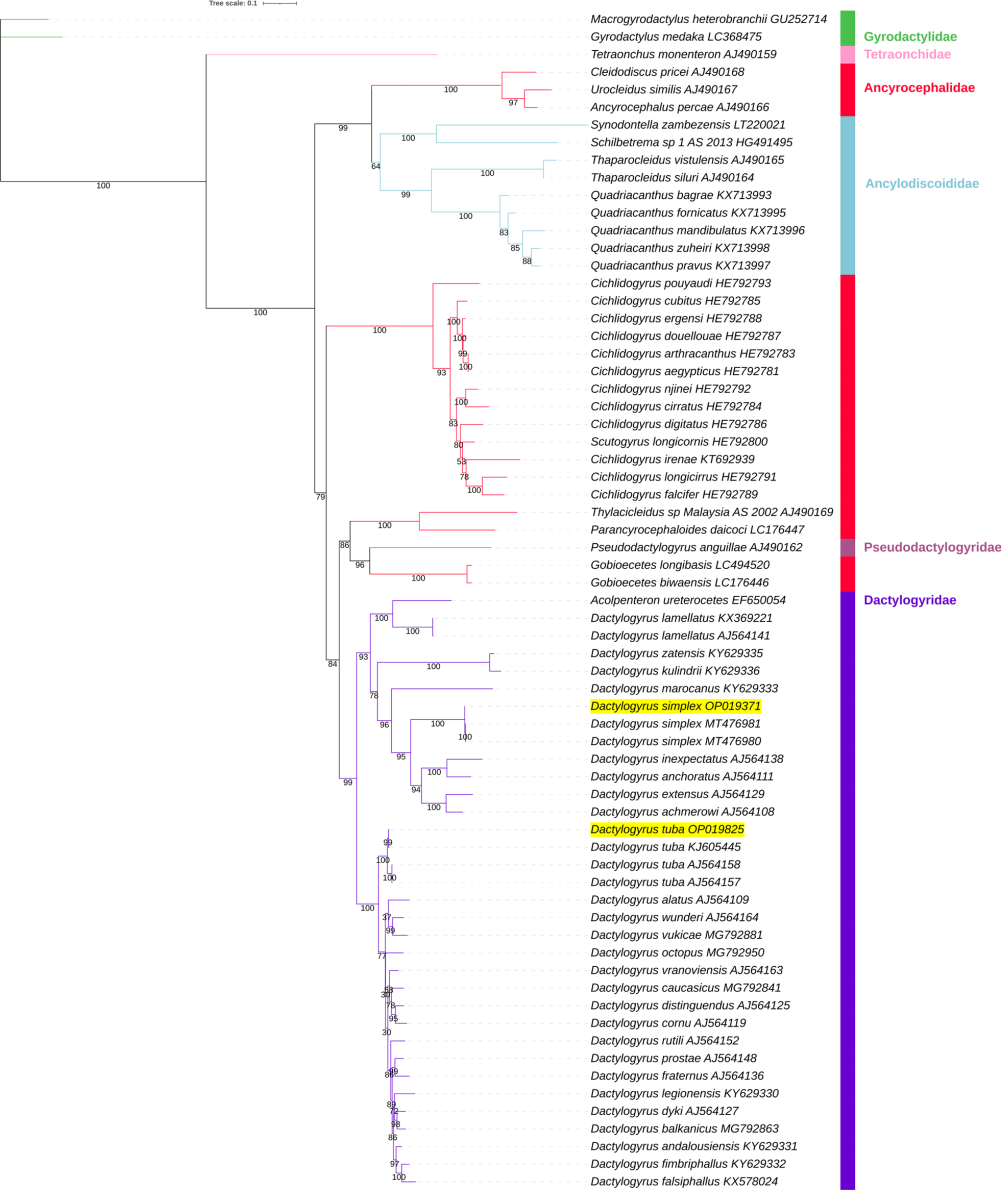
ITS1-based ML phylogenetic analysis of the Dactylogyridea. Two Gyrodactylidae species are outgroups. GenBank accession numbers are shown next to species names. Bootstrap support is shown next to nodes. The family level taxonomy is shown to the right. The two newly sequenced species are highlighted by a yellow background

**Fig. 9:**
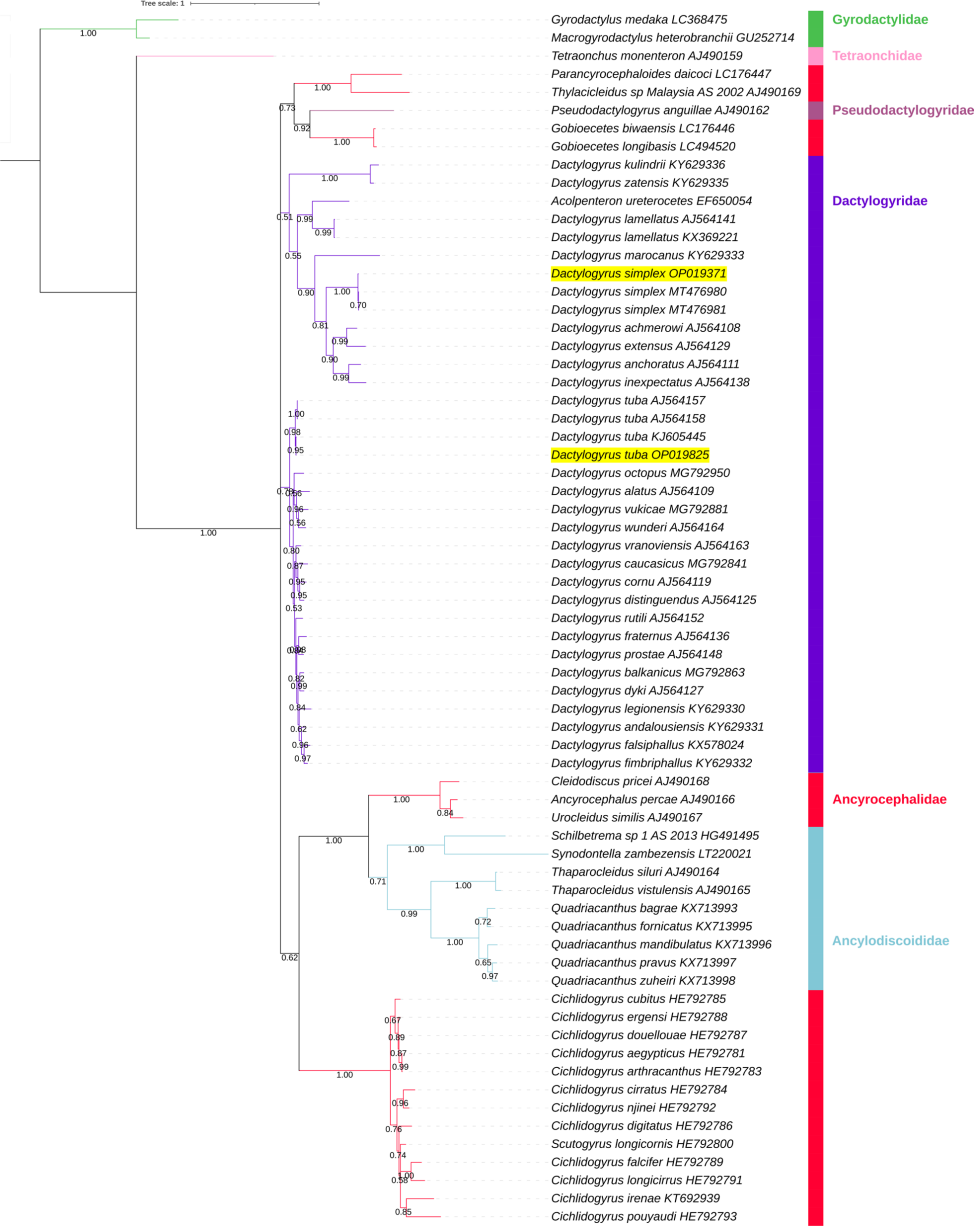
ITS1-based CAT-GTR phylogenetic analysis of the Dactylogyridea. Two Gyrodactylidae species are outgroups. GenBank accession numbers are shown next to species names. Bootstrap support is shown next to nodes. The family level taxonomy is shown to the right. The two newly sequenced species are highlighted by a yellow background

The newly generated ITS1 sequences (also comprising fragments of *18 s* and *5.8S*) were 1322 bp for *D. tuba* and 934 bp for *D. simplex*. We downloaded all homologous Dactylogyridae sequences from GenBank but kept only the ones exhibiting high coverage to ensure maximum resolution. BI and CAT-GTR analyses both converged. BI and ML analyses produced identical results (Fig. 8, Additional file 1: Figure S6). In this topology, Dactylogyridae and Ancylodiscoididae were monophyletic, while Ancyrocephalidae was deeply paraphyletic and split into three clades. One clade, comprised of *Cleidodiscus*, *Urocleidus* and *Ancyrocephalus*, clustered with Ancylodiscoididae (a sister-group relationship) with high support. None of these genera were available in the mitogenomic dataset, so we cannot make a meaningful comparison between the two datasets. Clade two comprised multiple *Cichlidogyrus* species, with *Scutogyrus* nested within, rendering the genus paraphyletic (again, assuming that this species was not misidentified). The third clade comprised *Thylacicleidus*, *Parancyrocephaloides* and *Gobiocetes*, with Pseudodactylogyridae (*Pseudodactylogyrus anguillae*) nested within. The family Dactylogyridae was split into two major clades. One comprised a group of nine *Dactylogyrus* species (including *D. simplex* and *D. lamellatus*) and *Acolpenteron ureterocetes*. The other comprised a group of 18 *Dactylogyrus* species (including *D. tuba*). Therefore, the family Dactylogyridae was monophyletic, but *Dactylogyrus* was split into two clades and rendered paraphyletic by *A. ureterocetes* (assuming that this species was not misidentified). The CAT-GTR phylogeny also produced the above three Ancyrocephalidae clades and the two Dactylogyridae clades, but the overall topology of these clades differed slightly; most notably, the two Dactylogyridae clades were polyphyletic (Fig. 9).

## Discussion

The two newly sequenced mitogenomes generally possessed standard features for neodermatan mitogenomes, including all genes being transcribed from the same strand and the missing *atp8*, which may be in the ground pattern of Platyhelminthes, but it is missing from all known Neodermata [7, 31, 48–50]. An intriguing feature of Monopisthocotylea is that it exhibits mitogenomes with rearranged gene orders, which is uncommon within the Neodermata [2]. However, the two newly sequenced mitogenomes exhibited an identical gene order, which was previously recognised as the ancestral architecture for Neodermata [2]. Both mitogenomes exhibited exceptionally low A + T base content within the dataset. Remarkably, in this aspect, the only other available congeneric species, *D. lamellatus*, was average among the Dactylogyridea. High A + T bias in flatworms was previously attributed to mutation, as opposed to selection [19], which suggests that these two species may evolve under lower mutational pressures. This is further supported by these two species exhibiting some of the lowest long-branch scores in the dataset.

The annotation of most genes was relatively straightforward, with *cytochrome b* (*cytb*) in *D. simplex* as an exception. Most of the available *cytb* orthologues use the TAG stop codon, but using this stop codon in *D. simplex* would create a 30-base 3’ end elongation compared to *D. tuba* and a large overlap of 25 bp with the downstream *nad4L*. As such large overlaps between PCGs other than *atp6-atp8* and *nad4L-nad4* pairs would be considered highly unusual [51], we opted to use an abbreviated T– stop codon, which merely caused a five-base abbreviation in *D. simplex* (1072 bp, compared to 1077 bp in *D. tuba*). We sequenced this section twice, but we failed to find evidence of a sequencing error (Additional file 1: Figure S7). We suspect that a single-base deletion mutation occurred near the 3’ end of this gene in its evolutionary history, because a single-base elongation mutation in its current sequence would cause a frameshift mutation that would make available two stop codons in the expected locations (the current 10-bp intergenic space between *cytb* and n*ad4L*): TAA and TAG. It would be interesting to corroborate this putative frameshift-causing deletion on a different conspecific specimen as well as closely related species. As per convention, *rrnS* was annotated to the adjacent downstream gene (*cox2*), which generated a 3’ extension of about 30 bp in *D. simplex* compared to most other orthologues.

Both species exhibited multiple large overlaps between genes. Whereas large overlaps comprising protein-coding genes (PCGs) are rare because of stringent evolutionary constraints, large overlaps between tRNA genes have been observed in multiple animal lineages [52]. However, the largest overlap of 28 bp did comprise two PCGs: *nad4* and *nad4L*. It was perfectly conserved in both species as well as in the third available congeneric mitogenome: *D. lamellatus* [7]. Therefore, it is highly evolutionarily conserved, and we have no reasons to suspect annotation artefacts. These two genes commonly overlap in other neodermatan species as well, but the size of the overlap varies [7]. This indicates that they are probably translated from an evolutionarily conserved bicistronic transcript, similar to *atp6/atp8* in many other animal mitogenomes [51, 53].

For congenerics, the two newly sequenced species exhibited remarkably low identities between orthologous gene pairs [54]. There are two possible explanations for this phenomenon: it may be reflective of the rapid molecular evolution of many flatworm lineages [22, 55–57] or it may indicate that the genus *Dactylogyrus* should be split into two different genera. Either way, the barcode identification thresholds proposed for other animal lineages [54] are almost certainly not applicable to this lineage. Indeed, it explains why our attempts to identify these two species using the BOLD database [58] were futile.

## Phylogenetic analysis

Compositional biases are known to affect phylogenetic reconstruction and other types of evolutionary studies [22, 23, 59]. As the two newly sequenced species exhibited atypical base composition within the dataset and most of the concatenated datasets failed to pass the compositional homogeneity test in IQ-TREE, we carefully designed phylogenetic analyses of this dataset with this confounding factor in mind. To test for topological stability, we conducted phylogenetic analyses using two standard algorithms on two different datasets: NUC and AAs. Furthermore, we applied the CAT-GTR algorithm, designed to account for compositional heterogeneity. A study has shown that this algorithm outperforms the standard methods when strong base composition biases produce phylogenetic artefacts [22]. Indeed, our analyses revealed a rather pervasive topological instability, with six analyses producing six different topologies. Intriguingly, the instability was largely confined to the Dactylogyridae/Ancyrocephalidae clade (and some in the Gyrodactylidae clade). Compared to the mitogenomic dataset, the ITS1 dataset had a reduced resolution in terms of the power of the marker, but an improved resolution in terms of the availability of data. As small markers are not suitable for deep phylogenies, we focused only on the order Dactylogyridea. Comparison with the mitogenomic results was rendered difficult by the incomplete overlap of species available for the two datasets.

The addition of two new Dactylogyridae mitogenomes allowed us to study the relationships between Dactylogyridae and Ancyrocephalidae using mitogenomic molecular data. Despite the instability of this clade, all six mitogenomic and three ITS1 analyses produced topologies that rendered Ancyrocephalidae paraphyletic. Considering all nine topologies, the most parsimonious solution to resolve the widespread paraphyly is to create a catch-all Dactylogyridae s.l. clade comprising the current Ancyrocephalidae, Ancylodiscoididae, Pseudodactylogyridae and Dactylogyridae sensu stricto families. The alternative would be to erect a large number of small families with questionable support. A similar conclusion was reached based on 18 homologous series of morphological characters [12]. The lineages included in these two studies did not exhibit a full overlap, so it is difficult to compare the results, but this indicates that morphological and molecular markers produce relatively congruent signals. Ancyrocephalidae was already introduced, and our analyses leave no doubt about its paraphyly. In mitogenomic analyses, the family Ancylodiscoididae Gusev, 1961, was consistently monophyletic and resolved as a closely related sister clade to Dactylogyridae + Ancyrocephalidae. ITS1 analyses supported this topology but indicated that several Ancyrocephalidae genera cluster with this family. Although mitogenomic data did not question the paraphyly of this family, all analyses indicate a very close relationship with other families in the Dactylogyridae s.l. Ancylodiscoididae was initially a subfamily of the Dactylogyridae [12], but later it was elevated to the family level based on several morphological characteristics [60]. However, this revision was soon contested based on molecular data (*28S* rRNA fragment), which indicated that this may be merely another subfamily of Dactylogyridae [13]. The family Pseudodactylogyridae Gusev, 1965, also has a similar history: first a genus (*Pseudodactylogyrus*) of Ancyrocephalinae, later elevated to the subfamily Pseudodactylogyrinae and finally further elevated to the family Pseudodactylogyridae [11]. A *28S*-based phylogenetic analysis resolved Pseudodactylogiridae as clustering with some *Dactylogyrus* species and *Ancyrocephalus morgundae* [13]. Mitogenomes remain unavailable for this family, so we can only rely on the ITS1 analyses to debate its status, which suggest that these four families may be valid subfamilies of the Dactylogyridae s.l. family if some genera of the highly paraphyletic Ancyrocephalinae were reassigned to other subfamilies. *Cleidodiscus*, *Urocleidus* and *Ancyrocephalus* should be reassigned to Ancylodiscoidinae. *Thylacicleidus*, *Parancyrocephaloides* and *Gobioecetes* should be reassigned to Pseudodactylogyrinae. The current Dactylogyridae s.s. appear monophyletic, so they could be all assigned to Dactylogyrinae. Finally, the subfamily Ancyrocephalinae s.s. would then comprise *Cichlidogyrus* and *Scutogyrus* species. As the latter was nested within the former in both mitogenomic and ITS1 analyses, there is strong evidence that these two should be synonymised. This also makes it questionable whether Ancyrocephalinae deserves a subfamily status.

All three ITS1 analyses indicate that the genus *Dactylogyrus* may be paraphyletic and split into two distinct clades: one comprising a group of 9 *Dactylogyrus* species (including *D. simplex* and *D. lamellatus*) and *Acolpenteron ureterocetes* and the other comprising a group of 18 *Dactylogyrus* species (including *D. tuba*). However, mitogenomic data did not support this, as *D. simplex* and *D. tuba* clustered together in all four ML and BI analyses. As both species had unusual base composition biases within the dataset, this may be an artefact of two species with homoplastic base compositions clustering together [22, 61]. This hypothesis was supported by both CAT-GTR analyses, where *D. tuba* clustered with *D. lamellatus*. However, gene orders and base composition analyses indicate that *D. simplex* and *D. tuba* are more closely related to each other than to *D. lamellatus*. Therefore, different parameters and datasets produce multiple conflicting hypotheses for the topology of this genus, so this issue should be studied with utmost care before a revision is proposed.

Regarding other taxa, this study offers further evidence that mitogenomic data consistently support the validity of the Tetraonchoidea clade, and consistently they place it at the base of the Dactylogyridea + Capsalidea clade [62]. As there is partial support for this clade from *28S* rDNA data and morphological apomorphies [2], we can conclude that there is very strong molecular support for the validity of the Tetraonchoidea order. However, *P. inermis* exhibited a pronounced long-branch score, which indicates that we should exclude an LBA artefact before a revision is proposed. Our results consistently resolved the order Capsalidea as nested within the Dactylogyridea, thus rendering the latter paraphyletic. This was also observed in several previous mitochondrial phylogenomic studies [2, 5, 62, 63]. However, multiple studies that relied on morphological data and small molecular markers (*28S* rRNA) found that Capsalidea was resolved as evolutionarily distant from Dactylogyridea [4, 64–66]. As mitochondrial genomes are the most powerful marker so far applied to study this problem, this discrepancy may be attributable to the poor resolution of morphological and single-molecular marker data. This would indicate that Capsalidea should be reassigned to the order Dactylogyridea, where it may be given a suborder status. However, before a revision is proposed, it is necessary to exclude mitochondrial introgression in the evolutionary history of these orders. This may be achieved using a multilocus nuclear dataset. Although Gyrodactylidea was not the focus of our study, it deserves a brief mention that *Gyrodactylus* was rendered paraphyletic by *Gyrodactylus* sp. (MW464989) and *G. nyanzae* clustering outside the main *Gyrodactylus* clade. The former mitogenome is unpublished, so the paraphyly may be caused by a taxonomic misidentification, but *G. nyanzae* rendering the genus paraphyletic had been observed in the original publication [18], so this problem deserves further studies.

This study was hampered by the limited availability of data; for example, there are 12 valid genera in Dactylogyridae, but only one of them was represented in our dataset. We urge the sequencing of further Dactylogyridae mitogenomes. As our results clearly show that mitogenomic data produce a certain level of topological instability and phylogenetic artefacts, future studies should seek congruence between multiple types of markers before they propose further taxonomic revisions in this highly problematic group of parasitic flatworms.

## Supplementary Information

**Additional file 1: Figure S1.** The drawing of a *Dactylogyrus simplex* Bychowsky, 1936 specimen. **Figure S2.** The drawing of a *Dactylogyrus tuba* Linstow, 1878 specimen. **Figure S3.** The mitogenomic architecture of the studied Monopisthocotylea dataset. **Figure S4.** The NUC_ML mitochondrial phylogenomic analysis of the Monopisthocotylea. **Figure S5.** The NUC_ML_12PCGs mitochondrial phylogenomic analysis of the Monopisthocotylea. **Figure S6.** The ITS1-based BI phylogenetic analysis of the Dactylogyridea. **Figure S7.** Sequencing chromatogram comprising the ‘problematic’ segment comprising the 3’ end of *cytb*, 5’ end of *nad4L* and 10-bp intergenic space between them in *D. simplex*.

**Additional file 2: Worksheet A.** Comparative mitogenomics of Polyopisthocotylea. Taxonomy, mitogenome size and base composition. **Worksheet B.** Gene comparison in Dactylogyridea. Size, start/stop codons and skews.

## Data Availability

The two mitogenomes are available from GenBank under accession numbers OP058752 (*D. simplex*) and OP05873 (*T. tuba*), and the two ITS sequences under OP019371 (*D. simplex*) and OP019825 (*T. tuba*).

## Abbreviations

AAs: Dataset comprised of amino acid sequences of 12 PCGs
ASDSF: Average standard deviation of split frequencies
BI: Bayesian inference
ML: Maximum likelihood
ITS1: Internal transcribed spacer 1
LBA: Long-branch attraction
NUC: Dataset comprised of nucleotide sequences of 12 PCGs and two rRNAs
PCG: Protein-coding gene
rRNA: Ribosomal RNA
tRNA: Transport RNA
