# Supplementary material for: Mitochondrial phylogenomics provides conclusive evidence that the family Ancyrocephalidae is deeply paraphyletic
Source: Parasit Vectors. 2023 Mar 1;16:83. doi: 10.1186/s13071-023-05692-6 (PMC9979435; doi:10.1186/s13071-023-05692-6)
Supplement: Supplementary file 1 — Additional file 1: Figure S1. The drawing of a Dactylogyrus simplex Bychowsky, 1936 specimen. Figure S2. The drawing of a Dactylogyrus tuba Linstow, 1878 specimen. Figure S3. The mitogenomic architecture of the studied Monopisthocotylea dataset. Figure S4. The NUC_ML mitochondrial phylogenomic analysis of the Monopisthocotylea. Figure S5. The NUC_ML_12PCGs mitochondrial phylogenomic analysis of the Monopisthocotylea. Figure S6. The ITS1-based BI phylogenetic analysis of the Dactylogyridea. Figure S7. Sequencing chromatogram comprising the ‘problematic’ segment comprising the 3’ end of cytb, 5’ end of nad4L and 10-bp intergenic space between them in D. simplex. [file 13071_2023_5692_MOESM1_ESM.pdf]

Additional file 1 for:

Mitochondrial phylogenomics provides conclusive evidence that the family Ancyrocephalidae is deeply paraphyletic

Cui-Lan Hao, Nian-Wen Wei, Yan-Jun Liu, Cai-Xia Shi, Kadirden Arken, Cheng Yue

College of Veterinary Medicine, Xinjiang Agricultural University, Urumqi 830052, Xinjiang, China

*Correspondence: yuechengxnd@aliyun.com

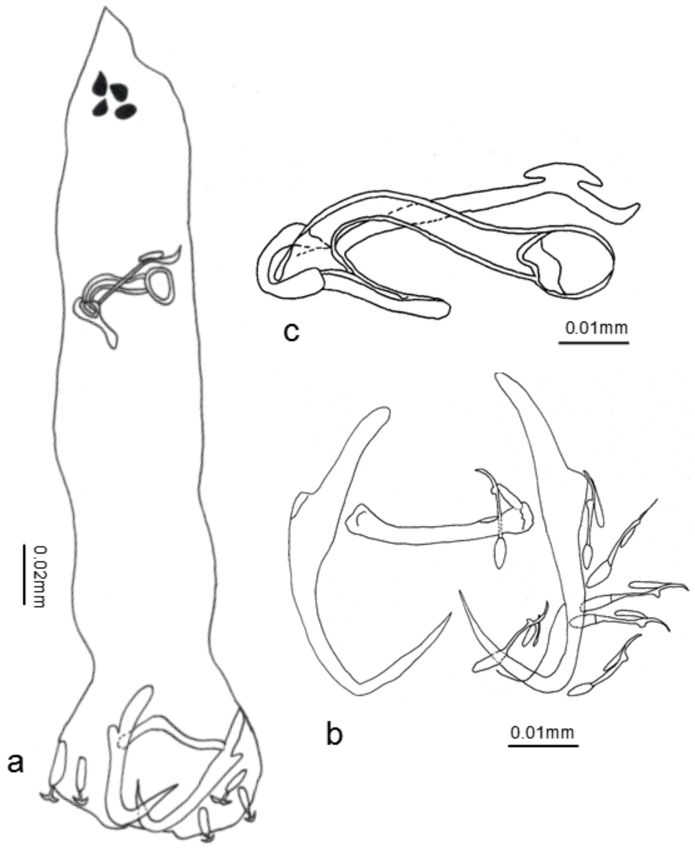

Figure S1. The drawing of a *Dactylogyrus simplex* Bychowsky, 1936 specimen. a) The entire specimen. b) Opisthaptor elements. c) Copulatory organ. Size bars are shown within the figure.

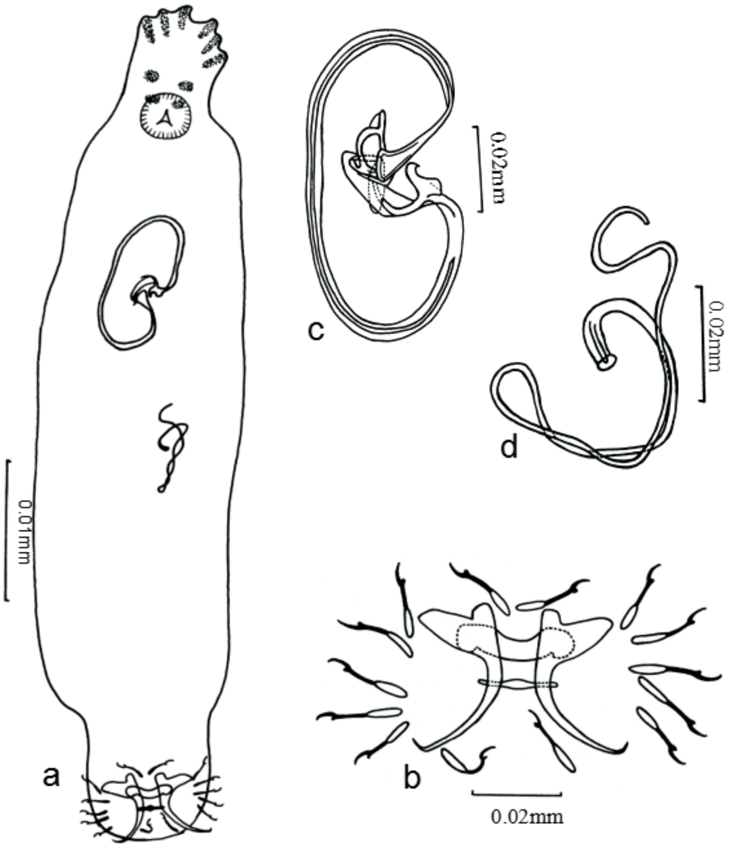

Figure S2. The drawing of a *Dactylogyrus tuba* Linstow, 1878 specimen. a) The entire specimen. b) Opisthaptor elements. c) Copulatory organ. d) Vagina. Size bars are shown within the figure.

.

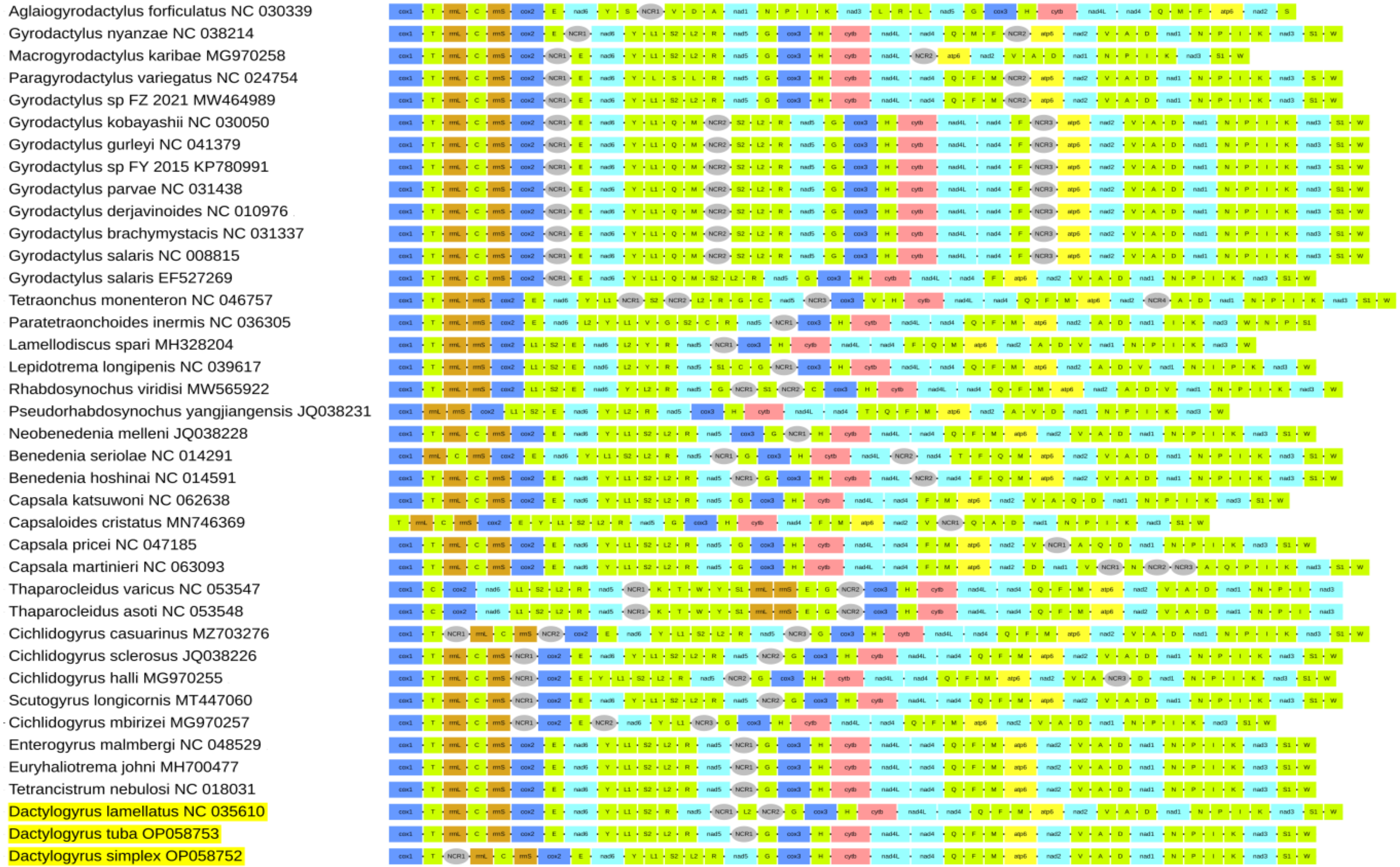
Aglaiogyrodactylus forficulatus NC 030339
Gyrodactylus nyanzae NC 038214
Macrogyrodactylus karibae MG970258
Paragyrodactylus variegatus NC 024754
Gyrodactylus sp FZ 2021 MW464989
Gyrodactylus kobayashii NC 030050
Gyrodactylus gurleyi NC 041379
Gyrodactylus sp FY 2015 KP780991
Gyrodactylus parvae NC 031438
Gyrodactylus derjavinoides NC 010976
Gyrodactylus brachymystacis NC 031337
Gyrodactylus salaris NC 008815
Gyrodactylus salaris EF527269
Tetraonchus monenteron NC 046757
Paratetraonchoides inermis NC 036305
Lamellodiscus spari MH328204
Lepidotrema longipenis NC 039617
Rhabdosynochus viridisi MW565922
Pseudorhabdosynochus yangjiangensis JQ038231
Neobenedenia melleni JQ038228
Benedenia seriolae NC 014291
Benedenia hoshinai NC 014591
Capsala katsuwoni NC 062638
Capsaloides cristatus MN746369
Capsala pricei NC 047185
Capsala martinieri NC 063093
Thaparocleidus varicus NC 053547
Thaparocleidus asoti NC 053548
Cichlidogyrus casuarinus MZ703276
Cichlidogyrus sclerosus JQ038226
Cichlidogyrus halli MG970255
Scutogyrus longicornis MT447060
Cichlidogyrus mbirizei MG970257
Enterogyrus malmbergi NC 048529
Euryhaliotrema johni MH700477
Tetrancistrum nebulosi NC 018031
Dactylogyrus lamellatus NC 035610
Dactylogyrus tuba OP058753
Dactylogyrus simplex OP058752

Figure S3. The mitogenomic architecture of the studied Monopisthocotylea dataset. The three *Dactylogyrus* mitogenomes are highlighted in yellow. Mitogenomes are shown linearized and rearranged to start with *cox1*.

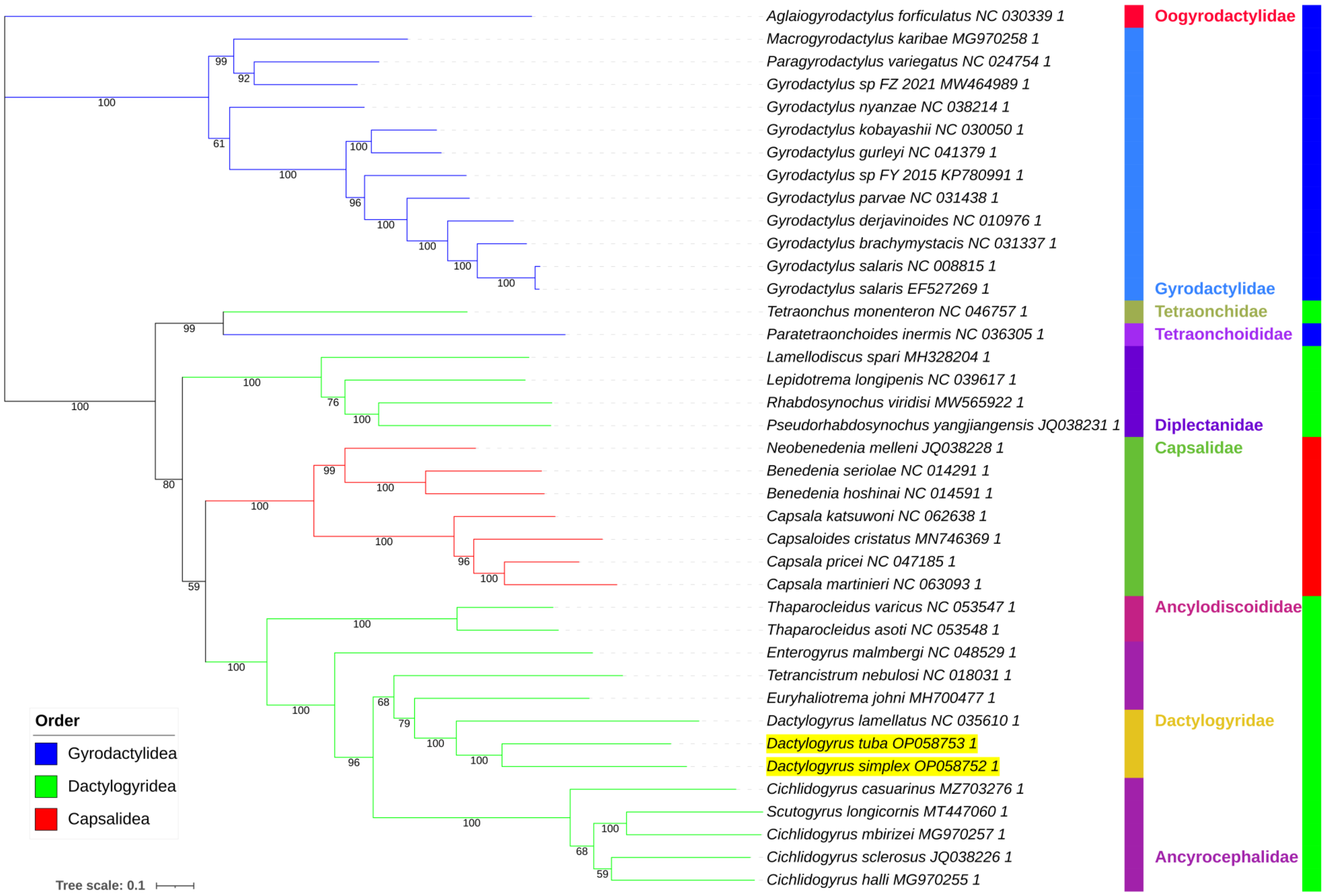
Aglaiogyrodactylus forficulatus NC 030339 1
Macrogyrodactylus karibae MG970258 1
Paragyrodactylus variegatus NC 024754 1
Gyrodactylus sp FZ 2021 MW464989 1
Gyrodactylus nyanzae NC 038214 1
Gyrodactylus kobayashii NC 030050 1
Gyrodactylus gurleyi NC 041379 1
Gyrodactylus sp FY 2015 KP780991 1
Gyrodactylus parvae NC 031438 1
Gyrodactylus derjavinoides NC 010976 1
Gyrodactylus brachymystacis NC 031337 1
Gyrodactylus salaris NC 008815 1
Gyrodactylus salaris EF527269 1
Tetraonchus monenteron NC 046757 1
Paratetraonchoides inermis NC 036305 1
Lamellodiscus spari MH328204 1
Lepidotrema longipenis NC 039617 1
Rhabdosynochus viridisi MW565922 1
Pseudorhabdosynochus yangjiangensis JQ038231 1
Neobenedenia melleni JQ038228 1
Benedenia seriolae NC 014291 1
Benedenia hoshinai NC 014591 1
Capsala katsuwoni NC 062638 1
Capsaloides cristatus MN746369 1
Capsala pricei NC 047185 1
Capsala martinieri NC 063093 1
Thaparocleidus varicus NC 053547 1
Thaparocleidus asoti NC 053548 1
Enterogyrus malmbergi NC 048529 1
Tetrancistrum nebulosi NC 018031 1
Euryhaliotrema johni MH700477 1
Dactylogyrus lamellatus NC 035610 1
Dactylogyrus tuba OP058753 1
Dactylogyrus simplex OP058752 1
Cichlidogyrus casuarinus MZ703276 1
Scutogyrus longicornis MT447060 1
Cichlidogyrus mbirizei MG970257 1
Cichlidogyrus sclerosus JQ038226 1
Cichlidogyrus halli MG970255 1
Oogyrodactylidae
Gyrodactylidae
Tetraonchidae
Tetraonchoididae
Diplectanidae
Capsalidae
Ancylodiscoididae
Dactylogyridae
Ancyrocephalidae
Order
Gyrodactylidea
Dactylogyridea
Capsalidea
Tree scale: 0.1

Figure S4. The NUC_ML mitochondrial phylogenomic analysis of the Monopisthocotylea. The NUC dataset comprised concatenated (partitioned) nucleotide sequences of 12 PCGs and 2 rRNAs. IQ-TREE was used to conduct the Maximum-Likelihood (ML) analysis. Bootstrap support is shown next to the nodes. The family-level taxonomy is shown to the right. Orders are indicated by a coloured strip and coloured branches, with the legend included in the figure. The two newly-sequenced species are highlighted by a yellow background.

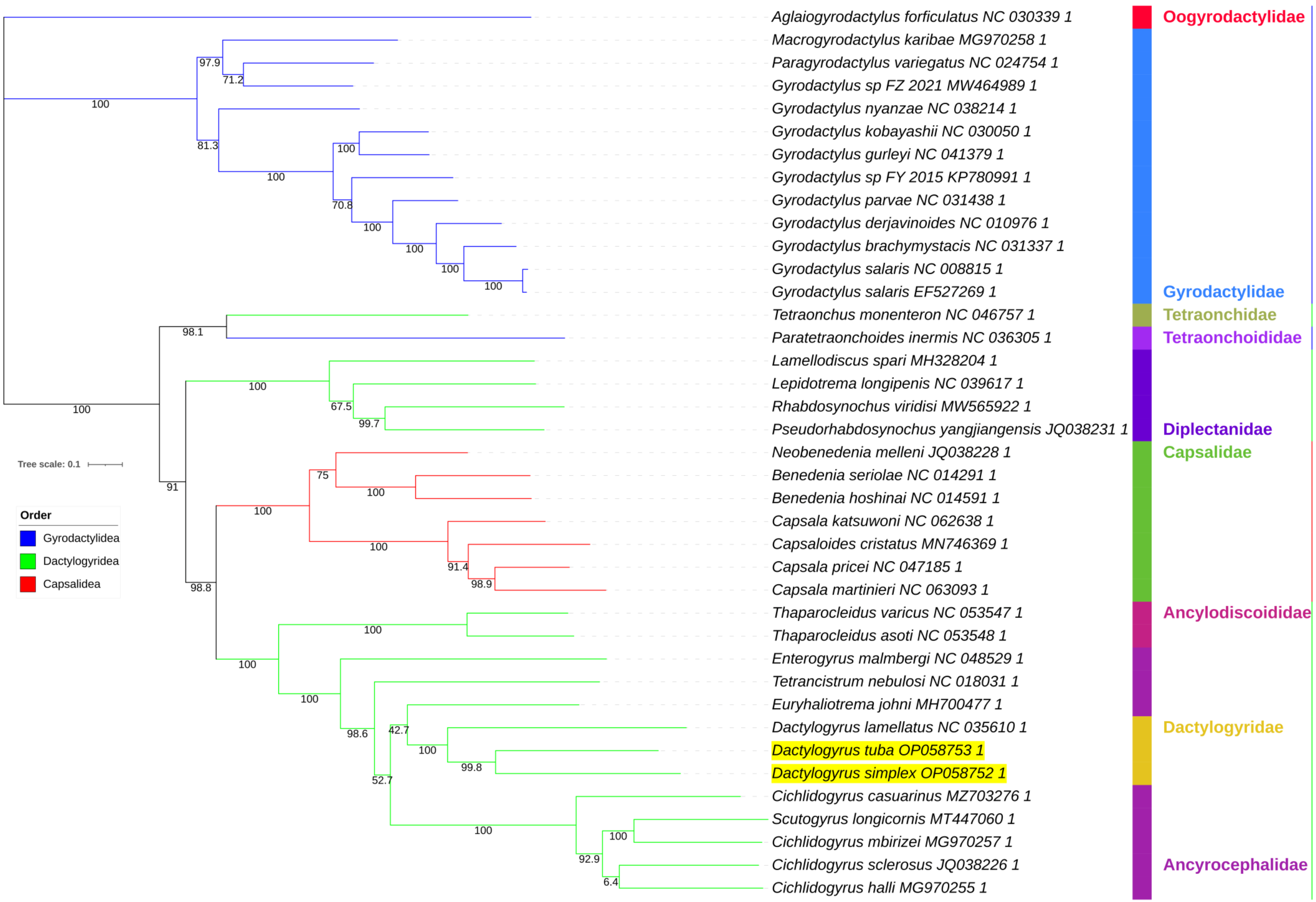
Aglaiogyrodactylus forficulatus NC 030339 1
Macrogyrodactylus karibae MG970258 1
Paragyrodactylus variegatus NC 024754 1
Gyrodactylus sp FZ 2021 MW464989 1
Gyrodactylus nyanzae NC 038214 1
Gyrodactylus kobayashii NC 030050 1
Gyrodactylus gurleyi NC 041379 1
Gyrodactylus sp FY 2015 KP780991 1
Gyrodactylus parvae NC 031438 1
Gyrodactylus derjavinoides NC 010976 1
Gyrodactylus brachymystacis NC 031337 1
Gyrodactylus salaris NC 008815 1
Gyrodactylus salaris EF527269 1
Tetraonchus monenteron NC 046757 1
Paratetraonchoides inermis NC 036305 1
Lamellodiscus spari MH328204 1
Lepidotrema longipenis NC 039617 1
Rhabdosynochus viridisi MW565922 1
Pseudorhabdosynochus yangjiangensis JQ038231 1
Neobenedenia melleni JQ038228 1
Benedenia seriolae NC 014291 1
Benedenia hoshinai NC 014591 1
Capsala katsuwoni NC 062638 1
Capsaloides cristatus MN746369 1
Capsala pricei NC 047185 1
Capsala martinieri NC 063093 1
Thaparocleidus varicus NC 053547 1
Thaparocleidus asoti NC 053548 1
Enterogyrus malmbergi NC 048529 1
Tetrancistrum nebulosi NC 018031 1
Euryhaliotrema johni MH700477 1
Dactylogyrus lamellatus NC 035610 1
Dactylogyrus tuba OP058753 1
Dactylogyrus simplex OP058752 1
Cichlidogyrus casuarinus MZ703276 1
Scutogyrus longicornis MT447060 1
Cichlidogyrus mbirizei MG970257 1
Cichlidogyrus sclerosus JQ038226 1
Cichlidogyrus halli MG970255 1
Oogyrodactylidae
Gyrodactylidae
Tetraonchidae
Tetraonchoididae
Diplectanidae
Capsalidae
Ancylodiscoididae
Dactylogyridae
Ancyrocephalidae
Tree scale: 0.1
Order
Gyrodactylidea
Dactylogyridea
Capsalidea
100
97.9
71.2
81.3
100
100
70.8
100
100
100
100
98.1
100
91
100
67.5
99.7
75
100
100
100
91.4
98.9
98.8
100
100
100
98.6
42.7
100
99.8
52.7
100
100
92.9
6.4

Figure S5. The NUC_ML_12PCGs mitochondrial phylogenomic analysis of the Monopisthocotylea. The NUC dataset comprised concatenated (partitioned) nucleotide sequences of 12 PCGs (2 rRNAs were not included). For other details please see Figure S4.

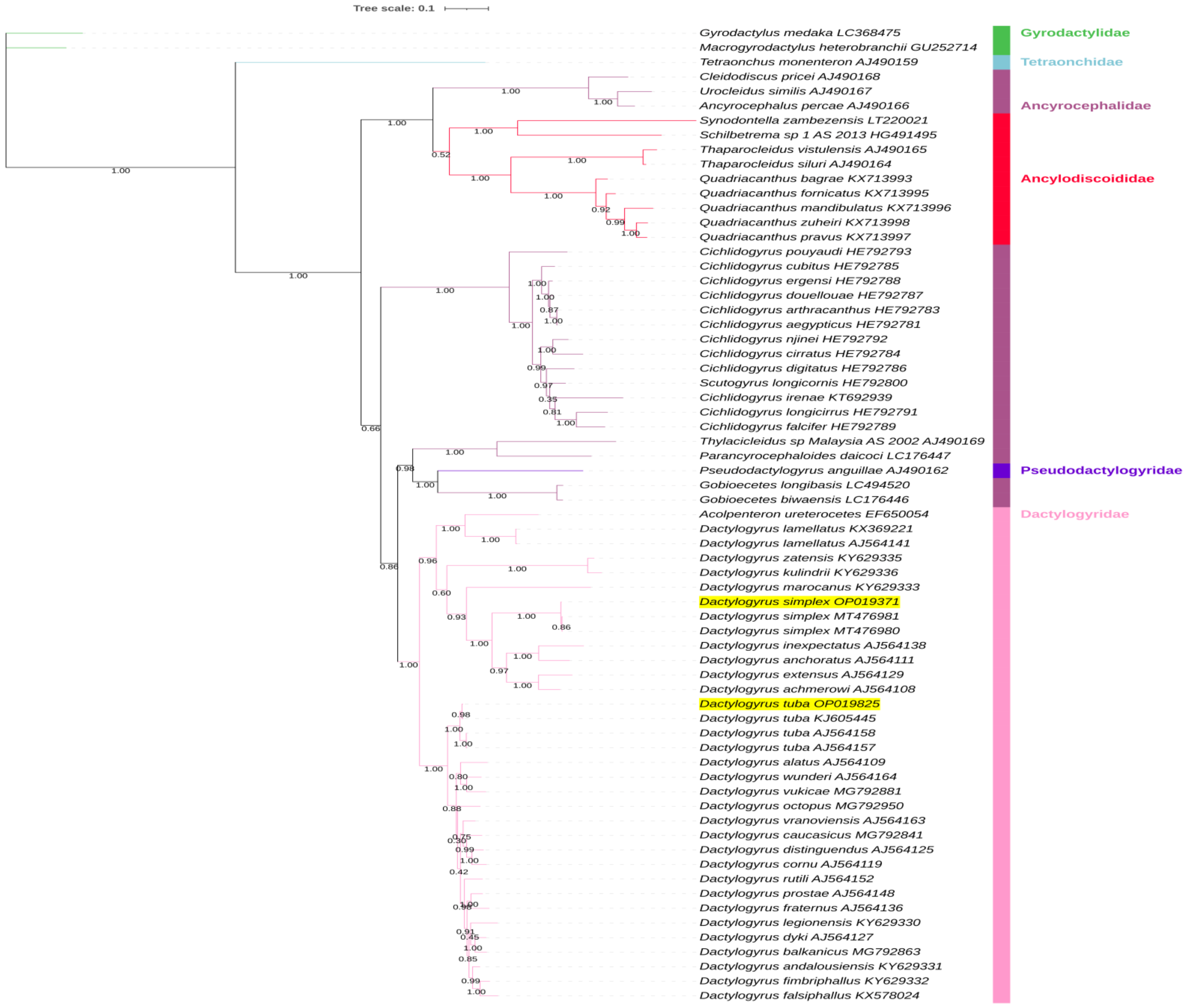
Tree scale: 0.1
Gyrodactylus medaka LC368475
Macrogyrodactylus heterobranchii GU252714
Tetraonchus monenteron AJ490159
Cleidodiscus pricei AJ490168
Urocleidus similis AJ490167
Ancyrocephalus percae AJ490166
Synodontella zambezensis LT220021
Schilbetrema sp 1 AS 2013 HG491495
Thaparocleidus vistulensis AJ490165
Thaparocleidus siluri AJ490164
Quadriacanthus bagrae KX713993
Quadriacanthus fornicatus KX713995
Quadriacanthus mandibulatus KX713996
Quadriacanthus zuheiri KX713998
Quadriacanthus pravus KX713997
Cichlidogyrus pouyaudi HE792793
Cichlidogyrus cubitus HE792785
Cichlidogyrus ergensi HE792788
Cichlidogyrus douellouae HE792787
Cichlidogyrus arthracanthus HE792783
Cichlidogyrus aegypticus HE792781
Cichlidogyrus njinei HE792792
Cichlidogyrus cirratus HE792784
Cichlidogyrus digitatus HE792786
Scutogyrus longicornis HE792800
Cichlidogyrus irenae KT692939
Cichlidogyrus longicirrus HE792791
Cichlidogyrus falcifer HE792789
Thylacicleidus sp Malaysia AS 2002 AJ490169
Parancyrocephaloides daicoci LC176447
Pseudodactylogyrus anguillae AJ490162
Gobioecetes longibasis LC494520
Gobioecetes biwaensis LC176446
Acolpenteron ureterocetes EF650054
Dactylogyrus lamellatus KX369221
Dactylogyrus lamellatus AJ564141
Dactylogyrus zatensis KY629335
Dactylogyrus kulindrii KY629336
Dactylogyrus marocanus KY629333
Dactylogyrus simplex OP019371
Dactylogyrus simplex MT476981
Dactylogyrus simplex MT476980
Dactylogyrus inexpectatus AJ564138
Dactylogyrus anchoratus AJ564111
Dactylogyrus extensus AJ564129
Dactylogyrus achmerowi AJ564108
Dactylogyrus tuba OP019825
Dactylogyrus tuba KJ605445
Dactylogyrus tuba AJ564158
Dactylogyrus tuba AJ564157
Dactylogyrus alatus AJ564109
Dactylogyrus wunderi AJ564164
Dactylogyrus vukicae MG792881
Dactylogyrus octopus MG792950
Dactylogyrus vranoviensis AJ564163
Dactylogyrus caucasicus MG792841
Dactylogyrus distinguendus AJ564125
Dactylogyrus cornu AJ564119
Dactylogyrus rutili AJ564152
Dactylogyrus prostae AJ564148
Dactylogyrus fraternus AJ564136
Dactylogyrus legionensis KY629330
Dactylogyrus dyki AJ564127
Dactylogyrus balkanicus MG792863
Dactylogyrus andalousiensis KY629331
Dactylogyrus fimbriphallus KY629332
Dactylogyrus falsiphallus KX578024
Gyrodactylidae
Tetraonchidae
Ancyrocephalidae
Ancylodiscoididae
Pseudodactylogyridae
Dactylogyridae

Figure S6. The ITS1-based BI phylogenetic analysis of the Dactylogyridea. Two Gyrodactylidae species are outgroups. GenBank accession numbers are shown next to species names. Posterior probability support values are shown next to nodes. The family-level taxonomy is shown to the right. The two newly-sequenced species are highlighted by a yellow background.

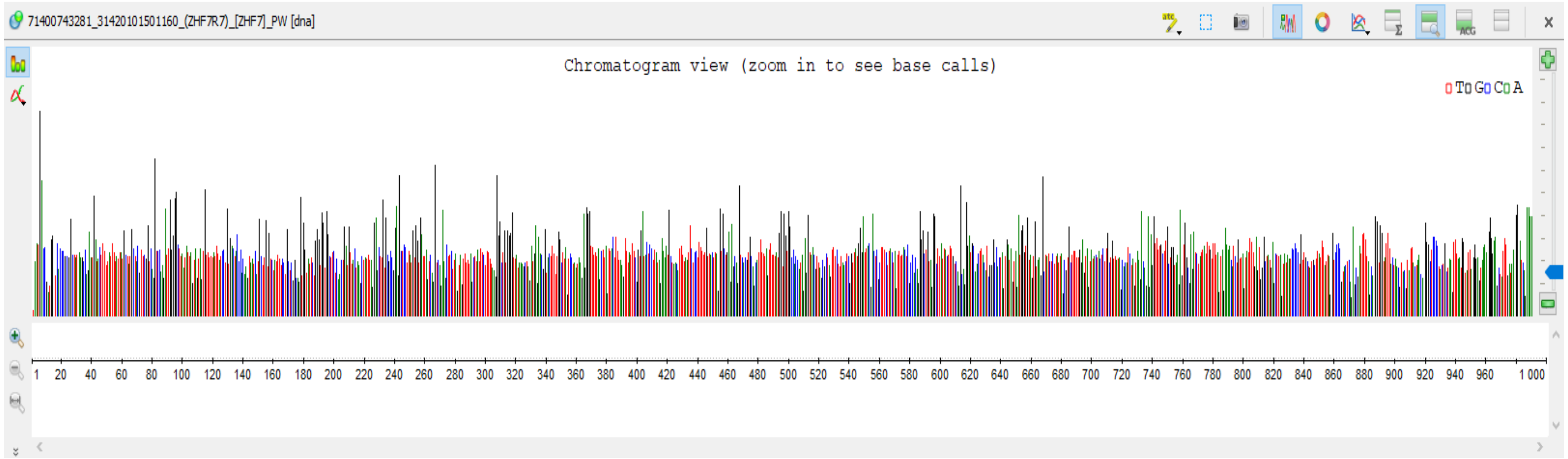

Figure S7. Sequencing chromatogram comprising ‘problematic’ the segment covering the 3’ end of *cytb*, 5’ end of n*ad4L*, and the 10 bp intergenic space between them in *D. simplex*.
